# Ivor Lewis esophagectomy patients are particularly vulnerable to respiratory impairment - a comparison to major lung resection

**DOI:** 10.1038/s41598-019-48234-w

**Published:** 2019-08-14

**Authors:** Martin Reichert, Magdalena Schistek, Florian Uhle, Christian Koch, Johannes Bodner, Matthias Hecker, Rüdiger Hörbelt, Veronika Grau, Winfried Padberg, Markus A. Weigand, Andreas Hecker

**Affiliations:** 1 Department of General, Visceral, Thoracic, Transplant and Pediatric Surgery, University Hospital of Giessen, Rudolf-Buchheim Strasse 7, D-35392 Giessen, Germany; 2 Department of Anesthesiology, Heidelberg University Hospital, Im Neuenheimer Feld 110, D-69120 Heidelberg, Germany; 3 Department of Anesthesiology, Intensive Care Medicine and Pain Therapy, University Hospital of Giessen, Rudolf-Buchheim Strasse 7, D-35392 Giessen, Germany; 4 Department of Thoracic Surgery, München Klinik Bogenhausen, Englschalkinger Strasse 77, D-81925 Munich, Germany; 5 Department of Pulmonary and Critical Care Medicine, University of Giessen and Marburg Lung Center (UGMLC), University Hospital of Giessen, Klinikstrasse 33, D-35392 Giessen, Germany; 6 Laboratory of Experimental Surgery, German Centre for Lung Research (DZL), Justus-Liebig-University Giessen, Feulgenstrasse 10-12, D-35392 Giessen, Germany

**Keywords:** Surgical oncology, Oesophageal cancer, Risk factors, Oesophagus

## Abstract

Pulmonary complications and a poor clinical outcome are common in response to transthoracic esophagectomy, but their etiology is not well understood. Clinical observation suggests that patients undergoing pulmonary resection, a surgical intervention with similarities to the thoracic part of esophagectomy, fare much better, but this has not been investigated in detail. A retrospective single-center analysis of 181 consecutive patients after right-sided thoracotomy for either Ivor Lewis esophagectomy (n = 83) or major pulmonary resection (n = 98) was performed. An oxygenation index <300 mm Hg was used to indicate respiratory impairment. When starting surgery, respiratory impairment was seen more frequently in patients undergoing major pulmonary resection compared to esophagectomy patients (p = 0.009). On postoperative days one to ten, however, esophagectomy caused higher rates of respiratory impairment (p < 0.05) resulting in a higher cumulative incidence of postoperative respiratory impairment for patients after esophagectomy (p < 0.001). Accordingly, esophagectomy patients were characterized by longer ventilation times (p < 0.0001), intensive care unit and total postoperative hospital stays (both p < 0.0001). In conclusion, the postoperative clinical course including respiratory impairment after Ivor Lewis esophagectomy is significantly worse than that after major pulmonary resection. A detailed investigation of the underlying causes is required to improve the outcome of esophagectomy.

## Introduction

Esophageal cancer is one of the leading cancer diagnoses with a high cancer-related mortality and a rapidly growing incidence over the past years world-wide^1^. The only curative treatment option in earlier, limited stages (Union internationale contre le cancer (UICC) – stages I and II) as well as in selected cases with a locally advanced disease (UICC stage III) is the subtotal resection of the esophagus, frequently following neoadjuvant and accompanied by adjuvant oncologic treatment modalities^1^. Nevertheless, the surgical therapy is challenging for the patients since it is associated with high rates of postoperative complications and morbidity. Overall, up to 60% of the patients experience any type of postoperative complication and the mortality rate of up to 14% after transthoracic esophagectomy for esophageal cancer is unacceptably high^2,3^. Especially pulmonary complications including atelectasis, pneumonia, pulmonary embolism, respiratory failure and acute respiratory distress syndrome (ARDS) are overrepresented after esophagectomy with an incidence ranging between 20% and 40% and consequently contribute to postoperative morbidity and even mortality^3–8^. After transthoracic esophagectomy, pneumonia, respiratory failure and ARDS rates accounted for 22%, 6% and 1.5%, respectively, in a large patient cohort reported by Zingg *et al*.^3^. As published recently, pulmonary complications after esophagectomy negatively impact not only on short-term clinical but also on long-term oncological patient outcome^3,9,10^. Pulmonary complications increase the time spent on the intensive care unit (ICU) and in the hospital, thus raising the overall health care costs after esophageal resections^3,9,11,12^. As it has been discussed by Molena *et al*., development of postoperative respiratory complications after esophagectomy is a multifactorial process^9^. Different hypotheses have been published on the pathophysiology of exceptional high pulmonary complication rates after esophagectomy with all kinds of consecutive respiratory impairment^9,13,14^.

However, these factors should resemble the pulmonary complication rates after conventional (open) major pulmonary resection (MPR). Similar to Ivor Lewis transthoracic esophagectomy (ILE), thoracotomy is used to access the thoracic cavity and single-lung ventilation is performed during MPR. Pulmonary complication rates after conventional open MPR are, however, considerably lower than those after esophagectomy^15,16^.

We conducted a single-center analysis to compare patients after right-sided thoracotomy for ILE with those after MPR, primarily focusing on the pulmonary outcome.

## Results

### Patients

Patient characteristics of both groups were similar regarding physical status, indicated by the ‘American society of Anesthesiologist’s classification of physical health’ (ASA) score, however, patients from the MPR-group suffered more frequently from chronic lung diseases (19.3% vs. 35.7%, p = 0.020). In contrast to the MPR-group, patients awaiting ILE underwent more frequently induction therapy of any kind (p < 0.0001). Indication for ILE was mainly primary malignancy (carcinoma of the esophagus in 98.8%). MPR was performed for malignancy in 88.8% (predominantly primary lung cancer) and benign diseases in 11.2% of the patients (lung volume reduction for emphysema (n = 1), inflammatory destroyed pulmonary lobe (n = 2), aspergilloma or tuberculoma (n = 4), and sequestration (n = 2), Table 1).

**Table 1:** Patient characteristics.

Variables	ILE	MPR	p-value
Male gender	68 (81.9%)	65 (66.3%)	0.019
Age [years]	63.0 (41–80)	62.0 (26–82)	0.631
BMI [kg/m²]	24.2 (15.6–41.3)	26.5 (15.9–39.0)	0.041
**ASA**			0.431
1	6 (7.2%)	9 (9.2%)	
2	40 (48.2%)	37 (37.8%)	
3	33 (39.8%)	49 (50.0%)	
4	4 (4.8%)	3 (3.1%)	
Chronic lung disease	16 (19.3%)	35 (35.7%)	0.020
Chronic kidney disease	6 (7.2%)	10 (10.2%)	0.602
**Induction therapy**			
Chemo	42 (50.6%)	8 (8.2%)	<0.0001
Radio	20 (24.1%)	2 (2.0%)	<0.0001
**Indication**			0.007
Malignancy	82 (98.8%)	87 (88.8%)	
Primary Tumor	82 (100%)	81 (93.1%)	
Metastasis	0	5 (5.7%)	
Lymphoma	0	1 (1.1%)	
Benign disease	1 (1.2%)	11 (11.2%)	

ILE = Ivor Lewis esophagectomy. MPR = major pulmonary resection. BMI = body mass index. ASA = American society of Anesthesiologist’s classification of physical health score.

### Surgery

The rate of intraoperative lymph node dissection was higher in the ILE-group (98.8% vs. 88.8%, p = 0.007), as was the total duration of the surgical procedure compared with patients from the MPR-group (306 (177–635) min vs. 180.5 (78–356) min, p < 0.0001) – although the rates of extended surgical procedures did not differ between both groups. Of note, the duration of the thoracic part of the two-stage ILE procedure was significantly shorter compared to the total duration of surgery in the MPR-group (142 (48–423) min vs. 180.5 (78–356) min, p < 0.0001, Table 2).

**Table 2:** Procedure characteristics.

Variables	ILE	MPR	p-value
Main procedure	Laparoscopy: 31 (37.3%) Laparotomy: 52 (62.7%) Gastric tube: 79 (95.2%) Colon interposition: 4 (4.8%)	Upper lobectomy: 45 (45.9%) Middle lobectomy: 5 (5.1%) Lower lobectomy: 33 (33.7%) Upper bilobectomy: 7 (7.1%) Lower bilobectomy: 8 (8.2%)	
Lymph node dissection	82 (98.8%)	87 (88.8%)	0.007
Relevant abdomino/thoracic extended procedures (additional to main procedure)	n = 17 (20.5%) Major lung resection: 3^#^ Minor lung resection: 5^#^ Lung decortication: 1 Minor liver resection: 3 Multivisceral resection: 1 Jejunum catheter: 3 Cholezystectomy: 1 Colon resection: 1 Others: 3^§^	n = 26 (26.5%) Sleeve resections, bronchoplasty: 11 Sublobar resection: 12^&^ Pleurectomy: 3 Decortication: 2 Chest wall resection: 1	0.3836
Duration of the thoracic part of Ivor Lewis procedure [min]	142 (48–423)^$^		<0.0001
Total duration of surgery [min]	306 (177–635)	180.5 (78–356)	<0.0001
Blood loss [ml]	600 (50–4800)*	500 (50–3000)	0.221
Peridural anesthesia	57 (68.7%)	49 (50%)	0.015

ILE = Ivor Lewis esophagectomy. MPR = major pulmonary resection. ^#^Including 3 lobectomies and 5 wedge resections. ^&^Including 3 segmentectomies and 9 wedge resections. ^§^Including appendectomy, resection of a soft tissue tumor and hemithyroidectomy. ^$^Not available retrospectively in 9 patients. *Not available retrospectively in 2 patients.

### Inflammation

Blood leukocytes on POD 0–2 were higher in patients of the MPR-group compared to the ILE-group. Later on POD 8-9, there was a tendency towards slightly higher leukocyte counts in ILE patients (p < 0.1). No differences in serum C-reactive protein (CRP) values were observed on POD 0-1. CRP levels tended higher after ILE compared to MPR on POD 2 and POD 5 (p < 0.1), whereas on POD 3-4 and POD 6-10 these differences between both patient groups were statistically significant (Table 3).

**Table 3:** Perioperative leukocyte counts and C-reactive protein levels.

Variables	ILE		MPR		p-value
Leukocytes [giga/l]		missing values		missing values	
POD 0 (on arrival at ICU)	8.6 (2.9–29.6)	1	13.1 (0.9–31.9)	3	<0.0001
POD 1	10.1 (3.6–71.0)	1	12.3 (4.2–30.3)	0	0.0001
POD 2	11.3 (1.8–24.6)	0	12.2 (3.8–114.0)	33	0.0488
POD 3	9.7 (1.9–34.1)	8	10.3 (3.6–21.3)	34	0.271
POD 4	8.3 (1.0–106.0)	14*	9.8 (2.5–22.9)	42	0.3052
POD 5	7.8 (3.9–21.5)	22*	9.5 (2.5–25.0)	52*	0.135
POD 6	9.3 (3.6–26.10)	26^#^	10.0 (3.8–21.5)	53*	0.992
POD 7	10.2 (2.9–29.0)	24^#^	10.4 (3.4–74.0)	47*	0.6968
POD 8	11.3 (3.4–33.3)	30^#^	9.7 (3.6–28.5)	61*	0.0927
POD 9	12.5 (4.5–49.7)	30^#^	10.8 (3.4–31.5)	68^#^	0.0747
POD 10	13.6 (4.2–38.7)	38^#^	11.65 (5.7–45.7)	74^#^	0.4021
**C-reactive protein [mg/l]**		**missing values**		**missing values**	
POD 0 (on arrival at ICU)	6.35 (0–80.1)	3	6.6 (0–238.7)	9	0.592
POD 1	92.79 (31.6–226.2)	1	87.25 (15.4–300.2)	0	0.24
POD 2	211.3 (82.7–359.4)	0	194.5 (46.8–390.7)	33	0.098
POD 3	219.2 (68.5–403.9)	8	166.7 (31.5–453.6)	34	0.001
POD 4	196.2 (30.1–410.0)	14*	153.4 (37.1–348.7)	43	0.019
POD 5	158.8 (37.1–539.1)	22*	109.1 (33.7–413.0)	52*	0.0776
POD 6	137.7 (24.1–423.2)	25^#^	74.4 (17.6–338.2)	53*	0.004
POD 7	147.7 (12.1–445.1)	24^#^	66.2 (3.9–408.8)	48*	0.0002
POD 8	152.8 (12.0–491.9)	31^#^	85.37 (22.9–270.2)	61*	0.002
POD 9	182.1 (19.0–446.9)	32^#^	73.1 (8.5–385.1)	68^#^	0.0008
POD 10	184.3 (4.9–387.3)	38^#^	97.8 (20.4–239.4)	74^#^	0.0111

ILE = Ivor Lewis esophagectomy. MPR = major pulmonary resection. *Including 1 death. ^#^Including 2 deaths.

### Respiratory impairment

The cumulative duration of perioperative mechanical ventilation (invasively and non-invasively) and cumulative postoperative stay on the ICU was longer in patients after ILE (both p < 0.0001). Accordingly, the rates of postoperative ventilation (p < 0.05 at POD 1-10), re-intubation (p = 0.0058) (independently from re-do surgery making re-intubation necessary), therapy with nitric oxide (p = 0.095), and of tracheotomy (p = 0.013) were higher in patients of the ILE-group indicating higher rates of severe respiratory impairment (Tables 4 and 5). Even higher rates of pneumonia defined by the ‘Uniform Pneumonia Score’^17^ (39.8% vs. 20.4%, p = 0.0053) and aspiration (10.8% vs. 1%, p = 0.006) were found in patients from the ILE-group (Table 5). These factors lead to higher rates of mechanical ventilation on POD 1-10 as well as longer cumulative duration of mechanical ventilation in patients after ILE compared to MPR (ILE-group: 17.93 (5–2280) h vs. MPR-group: 9.16 (3–701) h, p < 0.0001). Markedly, there were no differences in the rates of early postoperative mechanical ventilation (POD 0) as well as initial extubation success (during initial 12 h after surgery) between both groups (Tables 4, 5). The same holds true for intra- and early postoperative OI, where no obvious differences were found between both groups. However, since the rate of reduced OI (<300 mm Hg) was significantly higher initially in patients awaiting right-sided MPR (ILE: 17 patients vs. MPR: 38 patients, p = 0.009), the rates of reduced OI vice versa were significantly higher on all POD 1-10 in patients after ILE (overall 59 patients (71.1%) from the ILE-group and 35 patients (35.7%) from the MPR-group suffered from an OI < 300 mm Hg at least once during the observational period on POD 1-10, p < 0.0001, Table 5). Nevertheless, there was no difference concerning the clinical diagnosis of ARDS between both groups (ILE: 3 patients vs. MPR: 1 patient, p = 0.334).

**Table 4:** Perioperative results.

Variables	ILE	MPR	p-value
Total postoperative hospital stay [d]^$^	16 (9–68)	10 (6–87)	<0.0001
Cumulative postoperative stay on ICU [d]	4.64 (1–140)	0.95 (0–66)	<0.0001
Cumulative perioperative mechanical ventilation [h]	17.93 (5–2280)	9.16 (3–701)	<0.0001
**Rate of postoperative ventilation** ^**&**^			
POD 0 (on arrival at ICU)	66 (79.5%)	66 (67.3%)	0.093
Invasive	64	66	
Non-invasive	2	0	
POD 1	45 (54.2%)	23 (23.5%)	<0.0001
Invasive	41	22	
Non-invasive	4	1	
POD 2	17 (20.5%)	7 (7.1%)	0.0142
Invasive	9	5	
Non-invasive	8	2	
POD 3	19 (22.9%)	8 (8.2%)	0.0065
Invasive	13	7	
Non-invasive	6	1	
POD 4	20 (24.4%)	9 (9.2%)	0.0077
Invasive	16	7	
Non-invasive	4	2	
POD 5	19 (23.2%)	6 (6.2%)	0.0019
Invasive	16	4	
Non-invasive	3	2	
POD 6	20 (24.7%)	7 (7.2%)	0.0015
Invasive	18	5	
Non-invasive	2	2	
POD 7	24 (29.6%)	7 (7.2%)	0.0001
Invasive	20	5	
Non-invasive	4	2	
POD 8	22 (27.2%)	6 (6.2%)	0.0001
Invasive	20	5	
Non-invasive	2	1	
POD 9	19 (23.5%)	4 (4.2%)	0.0002
Invasive	18	3	
Non-invasive	1	1	
POD 10	21 (25.9%)	4 (4.2%)	<0.0001
Invasive	17	3	
Non-invasive	4	1	
Blood transfusion*	18 (21.7%)	18 (18.4%)	0.582
**Postoperative catecholamine therapy** ^**§,&**^			
POD 0	29 (34.9%)	17 (17.3%)	0.0098
POD 1	30 (36.1%)	14 (14.3%)	0.0009
POD 2	21 (25.3%)	6 (6.1%)	0.0003
POD 3	22 (26.5%)	8 (8.2%)	0.0012
POD 4	17 (20.7%)	7 (7.1%)	0.0086
POD 5	14 (17.1%)	4 (4.1%)	0.0053
POD 6	13 (16.0%)	5 (5.2%)	0.0232
POD 7	17 (21.0%)	4 (4.1%)	0.0007
POD 8	15 (18.5%)	4 (4.1%)	0.0027
POD 9	16 (19.8%)	2 (2.1%)	<0.0001
POD 10	17 (21.0%)	1 (1.0%)	<0.0001
Return to ICU	16 (19.3%)	12 (12.2%)	0.22
Re-do (revision) surgery	12 (14.5%)	6 (6.1%)	0.0811
Bronchial stump insufficiency after MPR/Anastomotic complications after ILE^ß^	13 (15.7%)	1 (1.0%)	
**Organ Failure**			
Liver	4 (4.8%)	1 (1.0%)	0.181
Kidney	5 (6.0%)	5 (5.2%)	1
Dialysis	5 (6.0%)	1 (1.0%)	0.095
In-hospital mortality^§^	11 (13.3%)	5 (5.1%)	0.07

ILE = Ivor Lewis esophagectomy. MPR = major pulmonary resection. POD = postoperative day. ICU = intensive care unit. $ excluding patients, who suffered from in-hospital mortality. *Within the first 24 hours after surgery, including blood transfusions, thrombocyte concentrates and fresh frozen plasma. ^§^Including arterenol and/or dobutamine. & Patients who died during POD 0–10 (n = 4, two in each group) were excluded from further analysis after their death. ß anastomotic complications, i.e. insufficiency and/or gastric tube necrosis requiring therapy (i.e. stent, endo-vacuum therapy or re-do surgery). § even exceeding 30-day mortality. The 30-day in-hospital mortality was 8.4% in the ILE-group and 5.1% in the MPR-group.

**Table 5:** Pulmonary outcome.

Variables	ILE		MPR		p-value
Pneumonia rate	33 (39.8%)		20 (20.4%)		0.0053
Pneumonia - diagnosis on POD	6 (0–25)^§^		3 (1–17)		0.0052
Tracheotomy	10 (12.0%)		2 (2.0%)		0.013
ECMO	4 (4.8%)		1 (1.0%)		0.181
NO therapy	3 (3.6%)		0		0.095
Aspiration	9 (10.8%)		1 (1.0%)		0.006
Initial extubation during first 12 h postoperatively	66 (79.5%)		88 (89.8%)		0.0614
Initial extubation ≥ POD 10	4 (4.8%)		1 (1.0%)		0.1809
Re-intubation^$^	22 (26.5%)		10 (10.2%)		0.0058
**Oxygenation Index**		**missing values**		**missing values**	
First intraoperative	416.8 (76.32–749.2)	1	425.8 (68.35–1219)	2	0.572
<300 mm Hg [n patients]	17 (20.7%)	1	38 (39.6%)		0.009
PEEP [mm Hg]	5 (1–12)		5 (0–13)		0.3
Last intraoperative	290.6 (55.79–655.7)		252.6 (43.33–637.9)		0.84
<300 mm Hg [n patients]	46 (55.4%)		56 (57.1%)		0.8808
PEEP [mm Hg]	5 (0–12)		5 (0–8)		0.207
POD 0 (on arrival at ICU)	381.6 (87.33–870.0)	0	401.4 (103.0–828.9)	5	0.188
<300 mm Hg [n patients]	27 (32.5)		18 (18.4%)		0.0379
POD 1	325.3 (113.8–843.3)	0	395.0 (128.0–1020)	14	0.0003
<300 mm Hg [n patients]	30 (36.1%)		15 (15.3%)		0.0017
POD 2	264.7 (83.17–685.0)	20	297.2 (112.3–483.3)	74	0.195
<300 mm Hg [n patients]	39 (47.0%)		13 (13.3%)		<0.0001
POD 3	259.9 (65.5–523.3)	38	263.3 (160.8–556.7)	80	0.784
<300 mm Hg [n patients]	29 (34.9%)		12 (12.2%)		0.0003
POD 4	236.0 (65.8–642.9)	44*	260.7 (110.7–646.7)	78	0.671
<300 mm Hg [n patients]	26 (31.7%)		17 (17.3%)		0.0346
POD 5	236.1 (101.9–642.0)	50*	284.1 (106.3–560.0)	83*	0.392
<300 mm Hg [n patients]	22 (26.8%)		10 (10.3%)		0.0057
POD 6	235.0 (106.6–419.5)	54^#^	317.7 (193.7–476.7)	84*	0.002
<300 mm Hg [n patients]	25 (30.9%)		6 (6.2%)		<0.0001
POD 7	227.1 (108.9–666.7)	59^#^	320.0 (158.0–790.5)	85*	0.0095
<300 mm Hg [n patients]	20 (24.7%)		6 (6.2%)		0.0006
POD 8	189.6 (95.33–685.0)	59^#^	420.0 (120.3–642.9)	87*	0.0042
<300 mm Hg [n patients]	22 (27.2%)		5 (5.2%)		<0.0001
POD 9	218.1 (75.29–533.2)	60^#^	219.9 (115.1–350.0)	91^#^	0.883
<300 mm Hg [n patients]	19 (23.5%)		6 (6.3%)		0.0019
POD 10	218.8 (80.32–523.3)	60^#^	238.7 (104.9–389.2)	91^#^	0.922
<300 mm Hg [n patients]	19 (23.5%)		6 (6.3%)		0.0019

ILE = Ivor Lewis esophagectomy. MPR = major pulmonary resection. POD = postoperative day. ECMO = extracorporal membrane oxygenation. NO = nitric oxide. ^§^One patient suffered from preoperative pneumonia. ^$^Independently from re-do (revision) surgery. Missing values for the postoperative oxygenation index come into account through discharge from ICU or death. The oxygenation index of patients intraoperatively as well as staying postoperatively on ICU is given in median(range). Patients not staying postoperatively on ICU are considered not to have any respiratory impairment (oxygenation index ≥300 mm Hg). *Including 1 death. ^#^Including 2 deaths.

As revealed by the Spearman’s Rho test, a correlation between total duration of surgery and early postoperative OI (POD 1-3), as well as cumulative duration of perioperative mechanical ventilation can be excluded (Table 6).

**Table 6:** Results of Spearman’s Rho rank correlation.

	Cumulative perioperative mechanical ventilation [h]	Oxygenation index				
		First intraoperative [mm Hg]	Last intraoperative [mm Hg]	POD 1 [mm Hg]	POD 2 [mm Hg]	POD 3 [mm Hg]
**Total duration of surgery [min]**						
ILE-group						
Correlation coefficient	0.186	0.118	0.084	−0.095	−0.171	−0.089
p-value (two-sided)	0.092	0.29	0.45	0.394	0.182	0.563
missing data	0	1	0	0	20	38
**Total duration of surgery [min]**						
MPR-group						
Correlation coefficient	0.141	0.02	0.94	−0.041	0.295	−0.053
p-value (two-sided)	0.166	0.849	0.358	0.712	0.162	0.836
missing data	0	2	0	14	74	80

ILE = Ivor Lewis esophagectomy. MPR = major pulmonary resection. The cumulative duration of mechanical ventilation as well as perioperative oxygenation indexes were independent from the duration of surgery.

**Figure 1:**
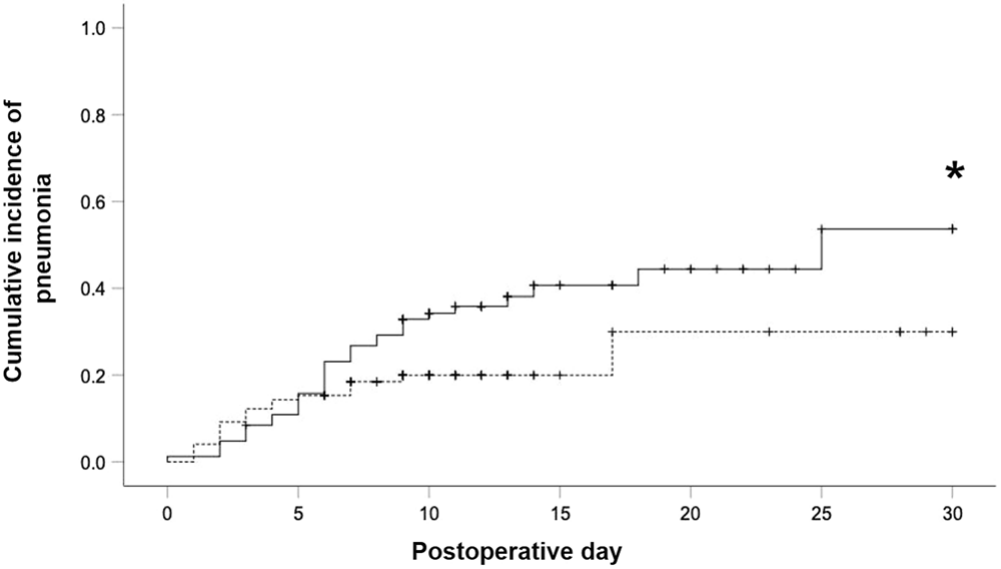
Kaplan-Meier estimation of cumulative incidence of postoperative pneumonia. Black line: Ivor Lewis esophagectomy (ILE)-group, n = 83 patients. Dashed line: Major pulmonary resection (MPR)-group, n = 98 patients. Patients who were discharged or died were censored from the analysis of cumulative incidence of postoperative pneumonia since the day of the event. Censored data are indicated in the figure by vertical ticks. *Indicates differences in the cumulative incidence of postoperative pneumonia between both groups at postoperative day 30 (p = 0.042).

**Figure 2:**
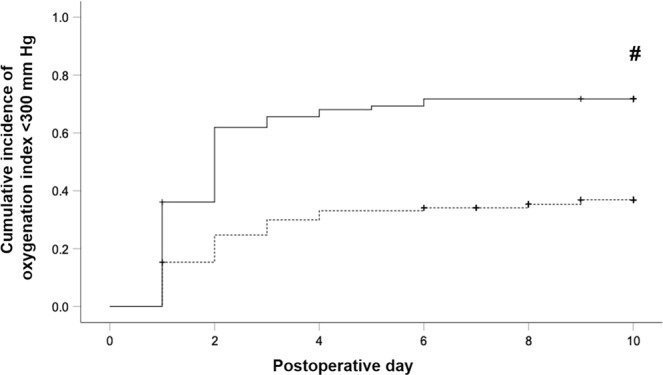
Kaplan-Meier estimation of cumulative incidence of postoperative oxygenation index <300 mm Hg indicating respiratory impairment. Black line: Ivor Lewis esophagectomy (ILE)-group, n = 83 patients. Dashed line: Major pulmonary resection (MPR)-group, n = 98 patients. Patients who were discharged, died or suffered from re-do (revision) surgery were censored from the analysis of cumulative incidence of postoperative reduced oxygenation index (<300 mm Hg) since the day of the event. Censored data are indicated in the figure by vertical ticks. An oxygenation index of <300 mm Hg on postoperative day 0 (arrival on ICU) was not judged as postoperative event. ^#^Indicates differences in the cumulative incidence of postoperative respiratory impairment (oxygenation index <300 mm HG) between both groups at postoperative day 10 (p < 0.001).

Kaplan-Meier analysis was performed to compare the cumulative incidences of postoperative pneumonia as well as postoperative respiratory impairment (OI < 300 mm Hg) over time. Pneumonia was more prevalent after ILE during POD 0–30 compared to right-sided thoracotomy for MPR (p = 0.042). This difference occurs beyond POD 10 (Fig. 1). In line with the differences observed in postoperative rates of reduced OI between both groups (Table 5), the cumulative incidence of respiratory impairment (OI < 300 mm Hg) differed significantly among the groups (p < 0.001) from POD 3 onwards (Fig. 2).

## Discussion

Respiratory complications are one major clinical problem in thoracic surgery, leading to unacceptably high rates of morbidity, disability and mortality^3–9,11^. Several studies have shown extraordinary high rates of pulmonary complications, especially of up to 40% pneumonia and up to 25% ARDS after ILE^7,12,18,19^. Perioperative atelectasis due to single-lung ventilation, postoperative pain after thoracotomy impairing respiratory physiology, manipulation and injury of the thoracic cavity and the lung during surgery, as well as potential laryngeal nerve injury caused by extended lymph node dissection or cervical esophageal preparation with an increased postoperative risk for aspiration were suggested to contribute to high pulmonary complication rates^9,13,14^. Except the obviously higher rate of aspiration due to delayed gastric emptying and a loss of the functional esophago-gastric junction in ILE-patients (10.8%, p = 0.006), these factors apply not only to ILE, but also to MPR with conventional thoracotomy. However, the previously reported pulmonary complication rates, especially for pneumonia (up to 6%^20–22^) and ARDS (approximately 4% in the early phase after anatomic lung resection^20,23^) are considerably lower after MPR compared with ILE, although – as shown in the present study – the rate of preexisting chronic pulmonary diseases is obviously higher in MPR patients.

In the present study, the complication rates after ILE correspond to those of previously reported patient cohorts^7^. Especially the rate of postoperative respiratory insufficiency leading to re-intubation of ILE patients is similar or even lower than those reported in the recent literature^7,24^. Nevertheless, pneumonia was assessed retrospectively through the “Uniform Pneumonia Score”^17^. The revised scoring system by Weijs *et al*.^17^ was herein applied in a slightly modified version with regard to the current “International Guidelines for the Management of Severe Sepsis and Septic Shock 2012”^25^ by using a body temperature ≥38.0 °C or ≤36.0 °C. This resulted in a high sensitivity of the scoring system for retrospective assessment of pneumonia in our patient cohort and a slightly higher rate of postoperative pneumonia of ILE and MPR patients (39.8% and 20.4%, respectively) than those reported in the recent literature^7,15,16,24^.

In addition to the clinical diagnoses of pneumonia and ARDS, the Horovitz OI (PaO_2_/FiO_2_) below 300 mm Hg was used as a sensitive indicator of respiratory impairment, irrespective of the underlying pulmonary disease^26^. Of note, the clinical diagnosis of ARDS (acute onset, bilateral pulmonary infiltrates, reduced OI^26^) was made in only a minority of our patients with an OI < 300 mm Hg. To the best of our knowledge, we are the first to describe an extraordinarily high cumulative incidence of respiratory impairment during POD 1–10 after ILE that is significantly higher than that of MPR patients (p < 0.001). In the light of a higher preoperative prevalence of preexisting chronic lung disease in MLR patients that is reflected by the first intraoperative OI value, the poor lung function of ILE patients on POD 1 and thereafter is even more striking. However, in both surgical approaches (ILE or MPR) the access to the thoracic cavity was the same standardized right-sided anterolateral thoracotomy and all patients underwent single-lung ventilation as well as most patients underwent disease-specific mediastinal lymph node dissection.

Development of postoperative respiratory complications after ILE and MPR is certainly a multifactorial process^9^: surgical tissue damage leads to an extensive inflammatory response, which may increase pulmonary endothelial permeability. Vascular leakage was previously suspected to contribute to acute lung injury and respiratory impairment after esophagectomy^27–30^. This would explain the early postoperative onset of ARDS after esophagectomy reported by Howells *et al*.^12^. However, pulmonary vascular leakage was not directly investigated in our study.

Among the various factors that can lead to respiratory impairment, transfusion-related acute lung injury is not expected to be responsible for the differences between ILE and MPR, because transfusion rates did not differ. The duration of single-lung ventilation was reported as a significant contributing factor for postoperative ARDS in the study by Tandon *et al*.^21^, while it did not seem to contribute to postoperative ARDS in the study by Morita *et al*.^31^. In our study, the total surgical procedure of ILE lasted longer than that of MPR. However, in contrast to the results of Tandon *et al*.^21^, the duration of the thoracic part of ILE was significantly shorter compared to MPR in our study (p < 0.0001). Hence, we can also exclude that differences in the duration of single-lung ventilation are responsible for the differences in respiratory impairment between ILE and MPR.

Also, the extent of tissue damage and the resulting release of pro-inflammatory damage-associated molecular patterns have to be considered. ILE is expected to generally cause more tissue damage compared to MPR, because, in addition to the surgical manipulations in the thorax, extensive surgery is performed in the abdominal cavity. However, MPR patients had higher leukocyte counts until POD 2, which might be explained by preoperative inflammation in this patient group. Further, CRP levels were similar on POD 1 and POD 2 and started to be higher in ILE patients as late as POD 3. Hence, the increased CRP levels in ILE patients are probably not directly related to differences in the extent or localization of surgery-associated trauma. However, we cannot fully exclude differences in trauma-associated inflammation, because unfortunately no pro-calcitonin or cytokine data were available in this retrospective study. Inflammatory markers should be carefully investigated in future prospective studies. In addition to trauma-associated inflammation caused by a more extensive surgery, the second operation site (laparotomy or laparoscopy) in ILE procedures could also contribute to pulmonary complications by increasing postoperative pain. For trans-thoracic procedures it is well known that minimally-invasive approaches result in reduced postoperative pain and lower pulmonary complication rates compared to conventional open thoracic surgery^16,31–33^. This has not only been shown for totally but also for hybrid minimally-invasive ILE procedures^34–37^. Briez *et al*. found that a hybrid minimally-invasive approach with laparoscopy for ILE procedures as well as pain management with epidural anesthesia are independent factors predicting against major postoperative pulmonary complications^35^. Also in a recently published well-controlled multicenter trial, Mariette *et al*. discussed reduced pain in response to their laparoscopic, hybrid minimally-invasive approach to ILE as the reason for a decreased rate of major pulmonary complications^36^. Further studies are needed to clarify this potential protective effect, especially with regard to perioperative oxygenation indexes.

The rate of neoadjuvant treatment clearly differed between both groups. The role of multimodal therapeutic approaches and especially induction therapies prior to ILE as a cause for an increased rate of postoperative pulmonary complications is still disputed. Reynolds *et al*. reported a higher rate of postoperative septic and pulmonary complications in patients, who underwent esophagectomy after induction therapy^38^, whereas Zingg *et al*. did not identify neoadjuvant treatment as a risk factor for postoperative pulmonary complications in their large patient cohort^3^. If alterations of the lung parenchyma including radiation pneumonitis and lung fibrosis, which can be observed in patients after irradiation of thoracic carcinomas^39^, play a role in the pathogenesis of perioperative respiratory impairment and postoperative pulmonary complications remains to be elucidated. However, an increased inflammatory status in ILE patients following radio-chemo induction should be reflected in an early elevation of leukocyte counts and CRP levels, which was obviously not the case.

Furthermore, the role of anastomotic complications in the development of respiratory impairment remains unclear. In this patient cohort only two patients of the ILE-group presented respiratory insufficiency (OI < 300 mm Hg) synchronously with the clinical diagnosis of anastomotic leakage requiring treatment. The other ILE patients either developed no respiratory impairment postoperatively (n = 1), presented a reduced OI (<300 mm Hg) metachronously (≥three days before anastomotic leakage, n = 4 patients) or were excluded from the Kaplan Meier analysis for a cumulative incidence of reduced postoperative OI due to re-do surgery. If the OI is sufficient to predict postoperative (pulmonary) complications in abdomino-thoracic surgery had – to the best of our knowledge – never been investigated before and should be evaluated in lager patient cohorts.

The surgical access route by thoracotomy is, however, notoriously a cause for postoperative pain, impairing postoperative breathing and respiratory physiology^9^. Previous reports have shown not only a reduction in pulmonary complications and pneumonia rates in patients undergoing (hybrid) minimally-invasive approaches for ILE or MPR as discussed above but also for abdominal-only transhiatal approaches for distal esophagectomy^3,13,14,32,33,40–43^. Especially after transhiatal esophagectomy, the rate of pulmonary complications is lower compared with the transthoracic approach^12–14,40^. However, the extent of mediastinal lymph node dissection and the location regarding the thoracic height of intrathoracic resection margins of the esophagus itself is technically limited by transhiatal esophageal surgery.

Nevertheless, the localization of esophageal resection margins with regard to the mediastinal level after transthoracic or transhiatal esophagectomy could explain the reported high rate of respiratory impairment in ILE patients as well as the observed major differences between both groups: some kind of neurogenic pulmonary damage has to be considered and discussed as a possible cause for the high rate of respiratory impairment after ILE. A dysbalance in the autonomic nervous system with a sympathetic hyperreaction is well-known in patients after brain damage, including the medulla and hypothalamic injury leading to neurogenic derived pulmonary edema formation by pulmonary vasculature constriction and pressure overload^44–46^. Vice versa, it had been shown in animal experiments that formation of pulmonary edema was either caused by bilateral (cervical) vagotomy^44^ or prevented by sympathetic denervation^46^. During ILE, surgeons usually transect the vagus nerve at the level, where the azygos vein joins the vena cava, which results in extensive or even complete vagal denervation of the lung^47^. Vagal branches carrying all parasympathetic and the vast majority of sensory fibers to the lung emerge from the vagal main trunk distally from this transection site down to the inferior margin of the main bronchus, so that this pathway is interrupted by cutting the vagus nerve more cranially^47^. Contrastingly, the sympathetic fibers innervating pulmonary structures, together with a few spinal sensory fibers, derive from the paravertebrally located sympathetic trunk, take a route along the bronchial artery^47,48^ and are obviously not injured to the same extent as parasympathetic and vagal sensory fibers during surgery. A vagus-sparing esophagectomy technique previously showed reductions in the rate of postoperative pneumonia, ARDS and delayed gastric emptying. This technique was, however, not combined with mediastinal lymph node assessment and is not suitable for oncologic surgical purposes with regard to oncological radicality^47,49^. A frequent and well-known postoperative clinical manifestation of bilateral truncal vagotomy during ILE is delayed gastric emptying and gastric outlet obstruction through pyloric denervation and consecutive spasm^50^. Even the high rate of postoperative atrial fibrillation after ILE might be partially caused by similar imbalances in the autonomic nervous system through higher mediastinal denervation^41,48,51^. We hypothesize, that a classical ILE with high thoracic esophageal resection margins and mediastinal lymph node dissection even leads to parasympathetic and sensory denervation of trachea, bronchi and pulmonary vasculature (especially pulmonary arteries) by vagotomy, the consecutive dysbalance of the autonomic nervous system innervating pulmonary structures would result in a higher sympathetic drive and thus may impair respiratory function and gas exchange widely analogous to the situation of neurogenic pulmonary edema.

Retrospectively conducted patient analyses like this study are only suited to generate hypotheses, which should be further tested in larger prospectively conducted trials with a high standardization of surgical procedures. Based on our findings, respiratory impairment beyond pulmonary complications after ILE need to be further evaluated, because these will have a major negative impact on the outcome of affected patients and prevention of postoperative pulmonary impairment is mandatory. This study shows, that beyond the surgical access route and the necessity of single-lung ventilation during surgery or inflammatory marker profile, other causes of lung function impairment after ILE must be considered. The impact of a dysbalanced autonomic nervous system on the perioperative pulmonary and cardiac function caused by ILE due to the concomitant vagotomy should therefore be examined in further prospectively trials, that should include e.g. a perioperative monitoring of heart rate variability, pulmonary arterial wedge pressure, pulmonary edema formation and immunological parameters.

## Materials and Methods

### Patients

The study was performed in accordance to the latest version of the Declaration of Helsinki and was approved by the local ethics committee of University of Giessen (AZ214/15). As this study was conducted retrospectively using routine patient data and as patient data were evaluated fully anonymized, written informed consent of the retrospectively included patients was waived by the local ethics committee in accordance to local legislation. 181 consecutive patients who underwent right-sided anterolateral thoracotomy for either ILE (n = 83) or MPR (lobectomy and bi-lobectomy, n = 98) were retrospectively identified during a seven-year period and included into this single-center analysis. Patients who underwent ILE together with any kind of pulmonary resection were included in the ILE-group. Patients who underwent left-sided thoracic surgery, right-sided anterolateral thoracotomy with minor lung resection (less than lobectomy: segmentectomy or (multiple) wedge resection) or pneumonectomy as well as patients who underwent transhiatal esophagectomy without thoracotomy were excluded from the analysis. All patients were treated according to the institutional standard-of-care. Patient data were analyzed retrospectively from the prospectively maintained institutional database. Patient data were analyzed during the first ten postoperative days (POD 0–10) regarding the Horovitz oxygenation index (OI), a key parameter to evaluate perioperative pulmonary function. OI-values were available for patients staying on ICU. Patients on a normal ward were excluded from analysis of OI-values and indicated in the tables as “missing values”. Consequently, discharge from the ICU was interpreted as absence of respiratory impairment or failure and an OI ≥ 300 mm Hg was assumed.

As described previously^26^, the OI was calculated as the ratio of the arterial pressure of oxygen (PaO_2_) and the fraction of inspired oxygen (FiO_2_) (PaO_2_/FiO_2_) at the beginning of mechanical ventilation (in most cases under double-lung ventilation), at the end of surgery, upon arrival at the ICU (POD 0), and on POD 1–10. If the PaO_2_ and FiO_2_ were assessed more than once a day, the first measurement of the day was used. According to the *Berlin classification*, an OI ≤ 300 mm Hg is an important criterion for the clinical definition of ARDS (mild: 201–300 mm Hg, moderate: 101–200 mm Hg and severe: ≤100 mm Hg)^26^. For mechanically ventilated patients (either invasively or non-invasively) the FiO_2_ was available. Patients who were not mechanically ventilated (invasively or non-invasively), a FiO_2_ of 30% was anticipated. An OI < 300 mm Hg was considered to indicate respiratory impairment or failure.

### Surgery

Right-sided anterolateral thoracotomy in the 4^th^ or 5^th^ intercostal space was the standard procedural access to the thoracic cavity for ILE as well as right-sided MPR. Regarding the local clinical standard ILE was performed as a synchronous two-stage procedure with laparotomy or laparoscopy for gastric tube building and gastric pull-up followed by an anterolateral thoracotomy on the right side to complete the subtotal esophagectomy and reconstruct the esophago-gastric continuity. Two-field lymph node dissection was performed as the standard procedure during oncologic esophagectomy, except in cases of cervical anastomosis, lymph node dissection was completed as a three field procedure following international recommendations^1^. The decision for laparoscopy or laparotomy was made based on the surgeon’s preferences and tumor stages. In cases of colon reconstruction, patients underwent primary laparotomy. Gastric tube was stapled from the small gastric curvature and anastomosis was usually made by using circular stapler devices. The time of the thoracic incision or of the intraoperative rearrangement of the patients into a lateral decubitus position for thoracotomy, respectively, was used to estimate retrospectively the time spent for of the transthoracic part of ILE, indicating the duration of single-lung ventilation during two-stage ILE.

For right-sided anatomic MPR a standard anterolateral thoracotomy in the 4^th^ or 5^th^ intercostal space was performed. Hilar structures were dissected and individually ligated, sutured or stapled. Systematic lymph node dissection in patients undergoing MPR for primary lung cancer was performed according to international recommendations^52,53^. Principles of the institutional standard in systematic lymph node dissection during lung cancer surgery were described previously in more detail in a patient cohort undergoing minimally-invasive oncologic pulmonary surgery by video-assisted thoracoscopy^54^.

In the postoperative phase, patients were treated by principles of an “enhanced recovery after surgery” protocol with extubation as soon as possible, early enteral nutrition and mobilization at the earliest convenience^55^. Usually, patients were monitored at the ICU after ILE or MPR for at least for one day (POD 1) and – if cardiorespiratorily stable – discharged from the ICU. One important clinical criterion for discharge from the ICU to a normal ward after major thoracic surgery is an adequate respiratory function. Patients on normal ward are classified as “normal oxygenation” with an OI ≥ 300 mm Hg (“no respiratory impairment” according to the ARDS definition^26^) to make them comparable to patients on ICU with regard to the rate of postoperative respiratory impairment or failure (OI < 300 mm Hg). Pneumonia was assessed and defined retrospectively in this study during the hospital stay using the “Uniform Pneumonia Score” introduced initially by van der Sluis *et al*. and revised by Weijs *et al*. as a standardized methodology to define pneumonia after esophagectomy^17,56^. However, apart from the revised “Uniform Pneumonia Score”, but according to current “International Guidelines for the Management of Severe Sepsis and Septic Shock 2012” we decided to use a body temperature ≥38.0 °C or ≤36.0 °C, respectively, as the threshold for pneumonia scoring in our study^17,25^.

### Statistical analyses

Statistical analyses were performed using GraphPad Prism (Version 5.00 for Windows, GraphPad Software, San Diego California USA, www.graphpad.com) for two group comparisons and SPSS (IBM SPSS Statistics for Windows, Version 24.0).

For descriptive statistics, data of both groups were analyzed using Fisher’s exact or Pearson’s X^2^ test for categorical data in cross-tabulation. Two group comparisons were performed by Mann-Whitney-U test. Patients who died were censored from analysis upon the day of death (indicated in the tables).

To determine statistical dependence, the total duration of surgery was correlated with the duration of cumulative (perioperative) mechanical ventilation as well as the intraoperative and postoperative (POD 1–3) OI by Spearman’s Rho rank correlation coefficient.

Cumulative incidences of postoperative pneumonia during POD 0–30 and postoperative respiratory impairment or failure (defined by an OI < 300 mm Hg) during POD 1–10 of patients after ILE or MPR were analyzed by Kaplan-Meier estimator. Patients who were discharged or died were censored from the analysis of cumulative incidences of postoperative pneumonia since the day the event. Patients who were discharged, died or underwent re-do surgery were censored from the analysis of cumulative incidences of postoperative reduced OI (<300 mm Hg) since the day of the event. Censored data are indicated in the figures by vertical ticks. An OI < 300 mm Hg on POD 0 (arrival on ICU) was not judged as postoperative event.

Data are given in tables as median and ranges; p-values ≤ 0.05 were considered to indicate statistical significance.

### Compliance with ethical standards

The data are collected, the manuscript is written and submitted in accordance to the COPE guidelines.

The acquisition of data and the study was formally approved by the local ethics committee of University of Giessen (AZ214/15). As this is a retrospective, fully anonymized patient data analysis, for this type of study formal written consent from the patients is waived by the local ethics committee in accordance to local legislation. All patients were treated by the current local standard of care. All procedures performed in studies involving human participants were in accordance with the ethical standards of the institutional and/or national research committee and with the 1964 Helsinki declaration and its later amendments or comparable ethical standards.
